# Analysis of EEG Characteristics of Drivers and Driving Safety in Undersea Tunnel

**DOI:** 10.3390/ijerph18189810

**Published:** 2021-09-17

**Authors:** Yongzheng Yang, Zhigang Du, Fangtong Jiao, Fuquan Pan

**Affiliations:** 1 School of Transportation and Logistics Engineering, Wuhan University of Technology, Wuhan 430063, China; yyongzheng@yeah.net (Y.Y.); zhig_du7@163.com (Z.D.); jiaofangtong@126.com (F.J.); 2 School of Civil and Environmental Engineering, Nanyang Technological University, 50# Nanyang Avenue, Singapore 639798, Singapore; 3 School of Mechanical and Automotive Engineering, Qingdao University of Technology, Qingdao 266520, China

**Keywords:** undersea tunnel, illuminance, longitudinal slope, EEG, correlation, driving safety

## Abstract

To study the influence of the driving environment of an undersea tunnel on driver EEG (electroencephalography) characteristics and driving safety, a real vehicle experiment was performed in the Qingdao Jiaozhou Bay Tunnel. The experimental data of the drivers’ real vehicle experiment were collected using an illuminance meter, EEG instrument, video recorder and other experimental equipment. The undersea tunnel is divided into different areas, and the distribution law of driving environment characteristics, EEG characteristics and vehicle speed characteristics is analyzed. The correlations between the driving environment characteristics, EEG characteristics and vehicle speed characteristics model the variables that pass the correlation test. The driving safety evaluation model of an undersea tunnel is established, and the driving safety in different areas of the undersea tunnel is evaluated. The results show that there are obvious differences in illumination, EEG power change rate, vehicle speed and other variables in different areas of the undersea tunnel. The driving environment characteristics are highly correlated with the β wave power change rate. The driving safety of different areas of the undersea tunnel from high to low is: upslope area, downslope area, exit area and entrance area. The study will provide a theoretical basis for the safe operation of the undersea tunnel.

## 1. Introduction

Undersea tunnel is a passage built under the sea for people and vehicles. It can change the traffic pattern and promote the social and economic development on both sides of the tunnel [1]. Undersea tunnel is a part of the urban road network. Compared with open road, the undersea tunnel has two obvious characteristics: complex light environment and longitudinal slope change. The path of the undersea tunnels enters the seabed from the land, passes under the seabed and moves outward to the land. This leads to a large longitudinal slope change throughout the undersea tunnel, which will have a negative impact on the driving safety [2]. At the same time, the entrance and exit of the undersea tunnel are the transition areas of sunlight. The illumination difference inside and outside the tunnel will lead to a “black hole effect” and a “white hole effect” at the entrance and exit of the undersea tunnel, and drivers will have temporary visual disturbances which will directly affect the driver’s driving ability [3]. Under the interaction of longitudinal slope and illumination changes, drivers will face greater tests in behavior and psychology, which will create a serious hidden danger for driving safety [4,5]. 

Undersea tunnel is an important traffic channel in urban traffic. In the undersea tunnel, the traffic volume is large, the speed is high, and the following distance is small [6]. Once a traffic accident occurs, it will often cause serious consequences, such as serial crashes, and will easily cause large-scale regional traffic congestion [7]. In addition, in case of congestion in the undersea tunnel, it will be difficult for rescue vehicles and to reach the injured to be rescued in time [8]. According to relevant research, the accident rate and severity of undersea tunnels are higher than that of ordinary highway tunnels and open road. The occurrence of traffic accidents in undersea tunnels is closely related to the light environment and longitudinal slope changes [9,10]. Therefore, studying the impact of the driving environment of the undersea tunnel on the psychology and behavior of drivers and exploring its inherent laws is very important and necessary for the safe operation of the undersea tunnel and the prevention of traffic accidents.

Some scholars have conducted related research on the psychology and behavior of drivers in highway tunnels. Jiao et al. [11] used the underwater tunnel as the research object, analyzed the influence of tunnel light environment change on driver eye movement parameters and found that the visual load in the entrance area is the largest. Du et al. [12] found that drivers mainly use the visual reference system of the tunnel environment to determine their own movement position, vehicle speed, direction and distance between vehicles, and proposes the use of sight guidance to optimize the tunnel driving environment, so as to enhance the driver’s sense of position in the tunnel. Pan et al. [13] analyzed the lane change behavior in different areas based on the road alignment in the undersea tunnel, and found that the risk of lane change in the downslope areas was the highest. Zhang et al. [14] analyzed the influence of illumination changes at the entrance and exit of the undersea tunnel on driving psychology, and the study found that drivers have obvious physiological fluctuations at the entrance areas, and the greater the illumination changes, the greater the physiological fluctuations of the driver. Zhang et al. [15] compared several underwater highway tunnels, and found that the longitudinal slope has a significant impact on road capacity, vehicle performance and drivers’ driving ability. The greater the longitudinal slope, the lower the road capacity, the worse the vehicle maneuverability.

The existing studies of highway tunnel traffic safety have shown that the dramatic change of the traffic environment is an important reason to induce traffic accidents [16]. In terms of research indicators, most of the existing studies use eye movement indicators, such as pupil area, blink frequency, eyelid closure and fixation duration, and ECG indicators such as heart rate, heart rate variability and heart rate growth rate, as well as skin conductance level, skin conductance response and other skin electrical indicators to analyze the physiological changes of drivers and then evaluate traffic safety in a specific environment [17,18,19]. Reimer [20] analyzed the relationship between the traffic environment and driving tasks during driving and found that driving safety is closely related to pupil area and blinking frequency. He et al. [21] found that the pupil area of drivers under different light environments in highway tunnels is different, and drastic changes in illumination can cause drivers to have a strong sense of tension, which in turn induces traffic accidents. Peng et al. [22] studied the changes in the driver’s field of view, fixation points and driving efficiency through simulated driving tests, and established a comprehensive evaluation model of eye movement characteristics. Feng et al. [23] established a model of the longitudinal slope, vehicle speed and heart rate variability rate and found that in the slope range of 3.5% to 4.0%, the driver’s heart rate variability was the highest, and the driving safety was the worst. Mo et al. [24] took the long tunnel as the research object, established the regression model between the driver’s heart rate growth rate and the mountain road linear index, and found that the driver’s heart rate growth rate was the highest and the driver was the most nervous when the downslope longitudinal slope was 3.5%.

At present, the research on tunnel traffic safety mainly focuses on the ordinary highway tunnels, while the research on undersea tunnel is less. At the same time, the characteristic quantity of research is mainly eye movement or ECG indicators. Compared with the ordinary highway tunnel, the undersea tunnel has a deeper depth, which will lead to more complex changes in the longitudinal slope of the undersea tunnel. Therefore, taking Qingdao Jiaozhou Bay Tunnel as the experimental tunnel to carry out the real vehicle experiment, and taking the EEG signal as the parameter, combined with the driving environment of Jiaozhou Bay Tunnel and the speed characteristics in the process of driving, the influence mechanism of the driving environment of the undersea tunnel on the driver is studied. It is expected to enrich the application of brain science research in the field of tunnel traffic, evaluate the traffic safety of different areas of the undersea tunnel, provide a theoretical basis for the operation and management of the undersea tunnel and ensure the traffic safety.

## 2. Experimental Scheme Design

### 2.1. Experimental Tunnel

The experimental tunnel is Qingdao Jiaozhou Bay Tunnel. Jiaozhou Bay Tunnel is located between Huangdao District and Qingdao District, with 6 lanes in two directions, and the maximum speed limit is 80 km/h. The total length of the tunnel is 7.8 km, of which the cross-sea section is 4.1 km, and the deepest section is 82.8 m below sea level. It is a typical V-shaped undersea tunnel [25].

**Figure 1:**
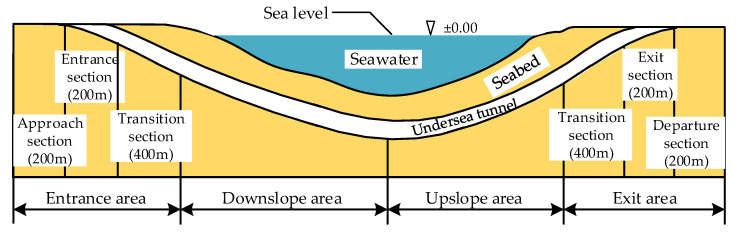
The area division of the undersea tunnel.

Based on the design specification of highway tunnels (JTG D70/2-2014, in China) and other documents [26,27,28], combined with the unique traffic conditions of the undersea tunnel, the experimental tunnel is divided into four parts: entrance area, downslope area, upslope area and exit area. The entrance area is divided into an approach section, entrance section and entrance transition section, and the exit area is divided into a departure section, exit section and exit transition section. The area division of the undersea tunnel is shown in Figure 1.

### 2.2. Experimental Equipment

The experimental vehicle is a family car in good condition. The illuminance measuring equipment is a TES-1339R professional illuminance meter, which has a measuring range of 0.01–9,999,900 lux, a resolution of 0.01 lux, a measurement accuracy of 3% and a sampling rate of 5 times/s. The EEG measurement equipment is an Emotiv EEG instrument with a measurement range of 0.2–45 Hz and the sampling rate is 258 times/s. The electrode placement method of the 10–20 international system is adopted. There are 16 electrode channels in total. The electrode channels cover 4 brain areas, of the frontal lobe, parietal lobe, temporal lobe and occipital lobe [29]. The vehicle speed-related data measuring equipment is a BWT901CL three-axis accelerometer. Other auxiliary equipment includes a video recorder fixed in the car and an automobile data recorder with time and speed recording functions.

### 2.3. Experiment Time and Driver

In order to avoid the interference of the traffic flow to the experiment drivers as much as possible, the experimental period is selected during the common traffic period (9 a.m. to 11 a.m. and 2 p.m. to 4 p.m.). The latitude of the Jiaozhou Bay Tunnel is 35 degrees north latitude. On a sunny day with good weather, the illuminance in this area is generally higher than 40,000 lux. Relevant research shows that [30] the tunnel entrance area is an accident-prone area, which is caused by the drastic changes in illumination at the entrance of the tunnel due to the shielding of tunnel shelter. The greater the illumination outside the tunnel, the more serious the “black hole effect” and “white hole effect” at the entrance and exit of the undersea tunnel, and the greater the threat to traffic safety. For the undersea tunnel, the worst environment is a sunny day with sufficient light. Therefore, to ensure the practicality of this research, bad weather such as rainy and foggy days were avoided during the experiment, and the experiment was carried out on sunny days.

A total of 26 drivers with a driving license participated in the experiment. Among them, 81% of drivers have more than two years of driving experience, 73% of drivers have driving experience in the undersea tunnel and the drivers are between 20 and 53 years old (34.5 ± 7.9). Meanwhile, there were 19 male drivers (73%) and 7 female drivers (27%). The age distribution and sex ratio are consistent with the current statistical characteristics of Chinese drivers [31]. Three days before the real vehicle experiment, all drivers were instructed to ensure a reasonable diet, good sleep and no drinking and medication behavior. In the real vehicle experiment, each experimental driver passed through and turned back through the Jiaozhou Bay Tunnel, and 50 groups of effective experimental data were obtained.

## 3. Results and Analysis

### 3.1. Analysis of Driving Environment Characteristics of the Undersea Tunnel

**Figure 2:**
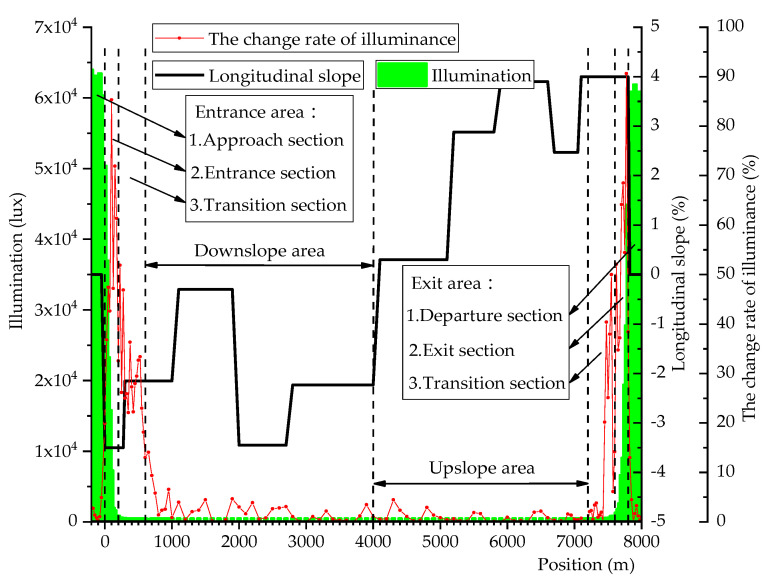
The distribution of illuminance and longitudinal slope of the undersea tunnel.

The illuminance data and longitudinal slope obtained from the experiment were processed, and the distribution of illuminance and longitudinal slope of the undersea tunnel is shown in Figure 2.

During the driving process, the driver’s ability to perceive the external environment is not only affected by the magnitude of the illuminance, but also depends on the intensity of the illuminance change [32]. In order to better analyze the influence of illumination change on drivers, based on the perception law of human vision to illumination, the illumination data was processed, and the illumination change rate which represents the speed of illumination change was obtained:

(1) $$\Delta E=\frac{\left|E_{2}-E_{1}\right|}{\max\left\{E_{1},E_{2}\right\}}\times 100\%$$

where Δ*E* is the change rate of illuminance (%), Δ*E*∈[0, 1]: the larger the Δ*E*, the more severe the illuminance changes, and the worse the safety, while the smaller the Δ*E*, the slower the illuminance changes, and the better the safety. *E*_2_ is the illuminance in the current unit time (lux), and *E*_1_ is the illuminance of the previous unit time (lux).

It can be seen from Figure 2 that the illumination in the entrance and exit areas of the undersea tunnel changed dramatically, from about 70,000 lux to about 100 lux, and the illumination value decreased by about 700 times. The change rate of illuminance (Δ*E*) also changed drastically: Δ*E* increased sharply to the maximum value, then decreased sharply, and finally stabilized at about 2%. In this process, the driver’s ability to perceive the driving environment decreases, and the probability of traffic accidents increases. In addition, the change area of Δ*E* is obviously larger than that of illumination, and the illumination change is mainly concentrated in the entrance and exit sections, while Δ*E* still has a higher value and a more dramatic change in the entrance transition section and exit transition section. According to the different longitudinal slopes, the Jiaozhou Bay Tunnel is divided into 12 sections, and the longitudinal slopes range from −4.00% to 4.00%.

### 3.2. Analysis of EEG Characteristics

According to the frequency, EEG signals can be divided into Δ wave, *θ* wave, *α* wave and *β* wave. Among them, Δ wave and *θ* wave are slow waves, and *α* wave and *β* wave are fast waves [33].

When studying the physiological state of drivers, EEG power is often used to characterize the driver’s brain activity [34]. Among them,* β* wave power (fast wave power index) and (*θ* + *α*)/*β* wave power (slow wave power index) are most sensitive to the changes of brain activity [35]. Using EEG instrument-supporting software and MATLAB software to perform a series of operations, such as EEG inspection, EEG segmentation, filtering, frequency segmentation, baseline calibration, artifact removal and feature extraction on the EEG dataset, the EEG power of each wave can be obtained. In order to better study the influence of the driving environment on the driver’s EEG signal in the undersea tunnel, the change rate of EEG power, which represents the change of a driver’s brain activity, was proposed, with reference to the calculation method of illumination change rate:

(2) $$\Delta P_{i}=\frac{\left|P_{i2}-P_{i1}\right|}{\max\left\{P_{i1},P_{i2}\right\}}\times 100\%$$

where Δ*P_i_* is the change rate of EEG power (%), *I* = Δ wave, *θ* wave, *α* wave, *β* wave, Δ*P_i_*∈[0, 1]: the larger the Δ*P_i_*, the more intense the EEG power changes, the more excited the brain and the more nervous the driver, while the smaller the Δ*P_i_*, the slower the EEG power changes and the calmer the brain. *P_i_*_2_ is the EEG power in the current unit time (10^−8^ V^2^/Hz), and *P_i_*_1_ is the EEG power of the previous unit time (10^−8^ V^2^/Hz).

**Figure 3:**
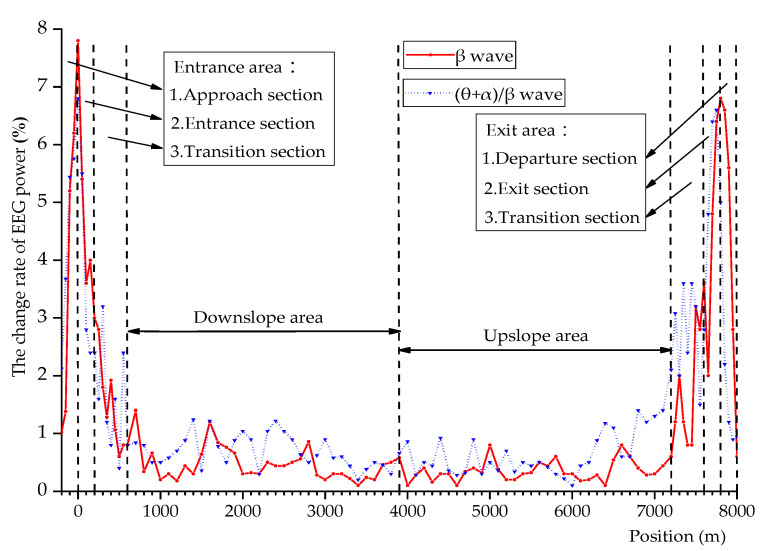
Distribution of the change rate of EEG (electroencephalography) power.

The EEG data obtained in the experiment was processed, and the distribution of the change rate of *β* wave power (Δ*P_β_*) and the change rate of (*θ* + *α*)/*β* wave power (Δ*P*_(*θ*+*α*)/*β*_) in the process of undersea tunnel driving is shown in Figure 3.

It can be seen from Figure 3 that the change trend of Δ*P_β_* and Δ*P*_(*θ*+*α*)/*β*_ is the same, and there is no obvious difference. At the entrance and exit area of the undersea tunnel, Δ*P_β_* rapidly increased to the maximum value, then rapidly decreased, and finally stabilized at about 0.5%. In this process, the driver’s brain is excited, and the driver’s psychological pressure and tension are high. Comparing Δ*P_β_* with Δ*E* in Figure 2, we can find that they have a high degree of similarity. The closer the driver is to the entrance and exit of the undersea tunnel, Δ*P_β_* and Δ*E* will fluctuate sharply and reach the maximum value. As the driver drives away from the entrance and exit of the undersea tunnel, Δ*P_β_* and Δ*E* will gradually decrease and tend to be stable.

### 3.3. Analysis of Speed Characteristics

**Figure 4:**
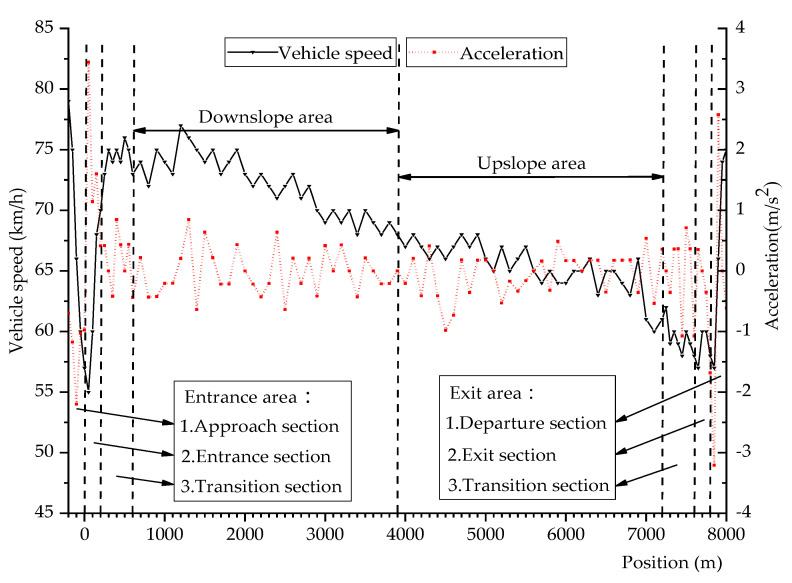
Distribution of the vehicle speed and acceleration.

Taking vehicle speed and acceleration as speed characteristic indicators, the vehicle speed and acceleration distribution during the driving process of the undersea tunnel are shown in Figure 4.

It can be seen from Figure 4 that when entering the entrance area of the undersea tunnel, the speed of the vehicle will decrease for a short time. As it gradually enters the interior of the undersea tunnel, the vehicle speed will gradually increase. In the downslope and upslope areas, the vehicle speed decreases gently, and the vehicle speed in the downslope area is significantly higher than that in the upslope area. In the exit area of the undersea tunnel, the speed is low. When the driver leaves the undersea tunnel, the speed will rise obviously. In terms of acceleration, during the whole driving process of the undersea tunnel, the acceleration of the vehicle is mainly between −3 and 3 m/s^2^, and the acceleration fluctuates smoothly. The acceleration at the entrance and exit areas of the undersea tunnel is slightly greater than that at the downslope and upslope areas.

## 4. Correlation Analysis and Modeling

### 4.1. Correlation Analysis

Correlation analysis is the analysis of two or more variable elements to measure the closeness of the correlation between the two variable elements. Correlation analysis can more accurately reflect the relationship between the characteristics of the driving environment, the characteristics of the speed and the characteristics of the driver’s EEG. The correlation formula is as follows [36]:

(3) $$r=\frac{\sum_{i=1}^{n_{0}}\left(X_{i}-\bar{X})(Y_{i}-\bar{Y}\right)}{\sqrt{\sum_{i=1}^{n_{0}}{\left(X_{i}-\bar{X}\right)}^{2}}\sqrt{\sum_{i=1}^{n_{0}}{\left(Y_{i}-\bar{Y}\right)}^{2}}}$$

where *r* is the correlation coefficient, |*r*|∈[0, 1]: the absolute value of *r* represents the size of the correlation, the larger the |*r*|, the stronger the correlation, and the smaller the |*r*|, the weaker the correlation. When |*r*| ≥ 0.8, the variables are highly correlated; when 0.8 > |*r*| ≥ 0.5, the variables are moderately correlated; when 0.5 > |*r*| ≥ 0.3, the variables are lowly correlated; when |*r*| > 0.3, the variables are not correlated. *n*_0_ is the number of samples. *X_i_* and *Y_i_* are the *i*-th value of the *X* and *Y* variables, respectively. $\bar{X}$ and $\bar{Y}$ are the average values of the *X* and *Y* variables, respectively.

Significance represents the credibility of the research results, and being able to pass the significance test is the prerequisite for the existence of a correlation between the research objects. Therefore, the correlation coefficient needs to be further tested for significance. First, we construct a statistic *t* with *r*:

(4) $$t=r\times \sqrt{\frac{n-2}{1-r^{2}}}$$

It can be proven mathematically that the degree of freedom of the statistic *t* is *t*-2 and conforms to the *t* distribution. Then, we can use the *t*-test to determine the significance of *r*. The significance level is 5%. When *p* > 0.05, it shows that *r* is not statistically significant; when *p* ≤ 0.05, it shows that *r* is statistically significant.

Correlation analysis was carried out on the characteristics of the driving environment of the undersea tunnel, the characteristics of the vehicle speed and the characteristics of the driver’s EEG. The analysis results are shown in Table 1.

It can be seen from Figure 2 that the illuminance only fluctuates in the entrance and exit areas of the undersea tunnel, while the illuminance is almost constant in the downslope and upslope areas. The changes of EEG signals and vehicle speed in the downslope and upslope areas of the undersea tunnel are independent of illumination, so the correlation test of the Δ*E* only considers the entrance and exit areas of the undersea tunnel. It can be seen from Table 1 that in the entrance and exit areas of the undersea tunnel, Δ*E* is highly correlated with Δ*P_β_*, moderately correlated with Δ*P*_(*θ*+*α*)/*β*_ and vehicle speed and lowly correlated with acceleration.

It can be seen from Figure 2 that the longitudinal slope changes throughout the undersea tunnel, so the correlation test of the longitudinal slope needs to consider the whole undersea tunnel. It can be seen from Table 1 that the longitudinal slope and Δ*P_β_* are highly correlated in the entrance area, downslope area and exit area of the undersea tunnel, and moderately correlated in the upslope area. Longitudinal slope and Δ*P*_(*θ*+*α*)/*β*_ are moderately correlated in the entrance area, downslope area and exit area of the undersea tunnel, and lowly correlated in the upslope area. The longitudinal slope is moderately related to the vehicle speed throughout the undersea tunnel. Longitudinal slope and acceleration are moderately correlated in the entrance area, lowly correlated in the upslope area and exit area and have no correlation in the downslope area.

According to the results of the correlation analysis, the characteristics with high or medium correlation were modeled.

### 4.2. Illumination Change Model

As the value of Δ*E* at different locations is quite different, the error generated when modeling it will also increase accordingly. In order to better analyze the relationship between Δ*E* and the change of EEG signal, the logarithm of Δ*E* was taken, and *ln*Δ*E* was taken as the illuminance change index, expressed by *q*, where the larger the *q*, the more severe the illuminance changes, and the smaller the *q*, the slower the illuminance change.

**Figure 5:**
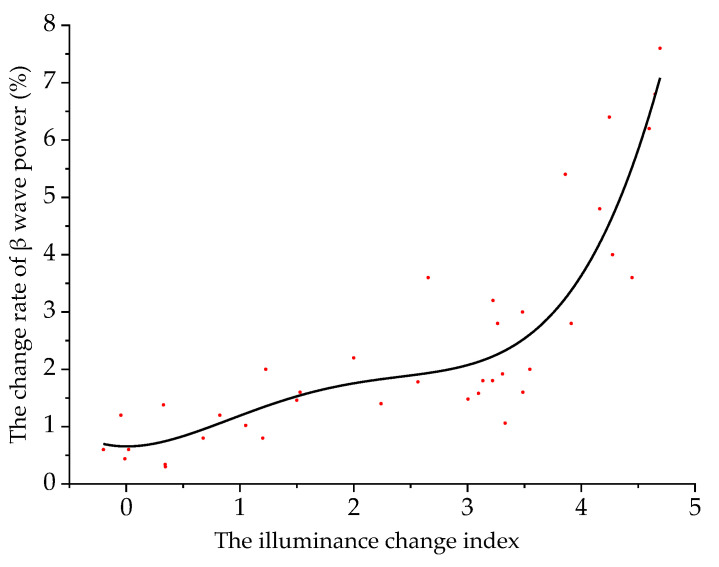
Fitting curve of illuminance change index and Δ*P_β_*.

Taking the illuminance change index as the independent variable and Δ*P_β_* as the dependent variable, the two were fitted. The scatter plot and the fitting curve of the illuminance change index and Δ*P_β_* are shown in Figure 5.

When a driver is driving in an undersea tunnel, the model of the illuminance change index and Δ*P_β_* is:

(5) $$y_{1}=0.66-0.08q+0.96q^{2}-0.49q^{3}+0.07q^{4}$$

where *y*_1_ is Δ*P_β_* (%), and *q* is the illuminance change index. The fitting determination coefficient *R*^2^ is 0.83, indicating that the reliability of the fitted model is good.

It can be seen from Figure 5 that the driver’s Δ*P_β_* will increase with the increase of the illuminance change index. The illuminance change index of 3.6 is the critical point of the model. When the illuminance change index is less than 3.6, as the illuminance change index increases, Δ*P_β_* increases slowly, the psychological pressure of the driver is low and the driving safety is good. When the illuminance change index is greater than 3.6, as the illuminance change index increases, Δ*P_β_* increases rapidly, the driver’s sense of tension increases and driving safety decreases.

**Figure 6:**
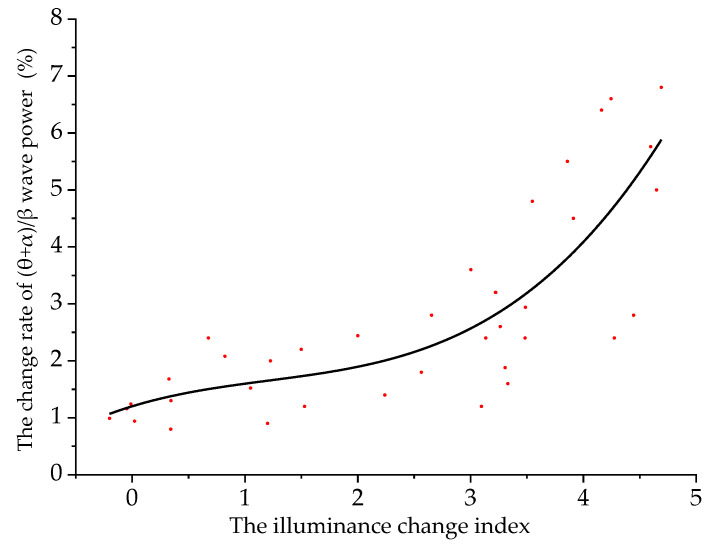
Fitting curve of illuminance change index and Δ*P*_(*θ*+*α*)/*β*_.

Taking the illuminance change index as the independent variable and Δ*P*_(*θ*+*α*)/*β*_ as the dependent variable, the two were fitted. The scatter plot and the fitting curve of the illuminance change index and Δ*P*_(*θ*+*α*)/*β*_ are shown in Figure 6.

When a driver is driving in an undersea tunnel, the model of the illuminance change index and Δ*P*_(*θ*+*α*)/*β*_ is:

(6) $$y_{2}=1.20+0.61q-0.29q^{2}+0.79q^{3}$$

where *y*_2_ is Δ*P*_(*θ*+*α*)/*β*_ (%), and *q* is the illuminance change index. The fitting determination coefficient *R*^2^ is 0.76, indicating that the reliability of the fitted model is good.

It can be seen from Figure 6 that with the increase of the illuminance change index, Δ*P*_(*θ*+*α*)/*β*_ increases steadily, the driver’s sense of tension gradually increases and the driving safety gradually decreases.

**Figure 7:**
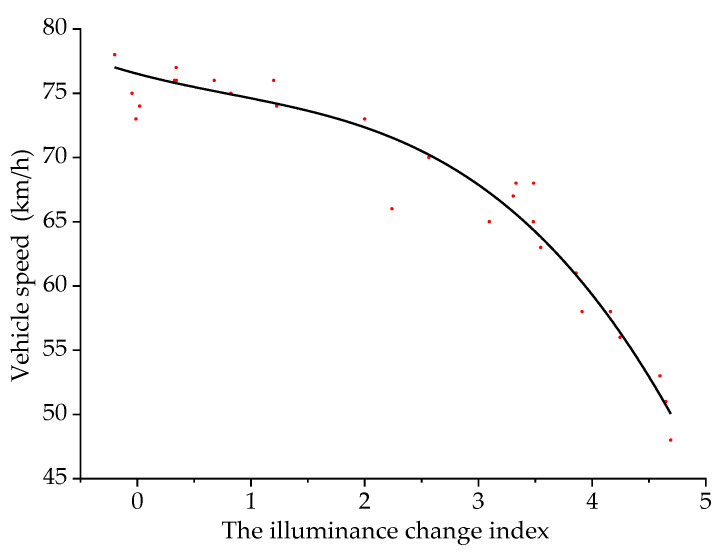
Fitting curve of illuminance change index and vehicle speed.

Taking the illuminance change index as the independent variable and the vehicle speed as the dependent variable, the two were fitted. The scatter plot and the fitting curve of the illuminance change index and the vehicle speed are shown in Figure 7.

When a driver is driving in an undersea tunnel, the model of the illuminance change index and the vehicle speed is:

(7) $$y_{3}=76.51-2.36q+0.76q^{2}-0.31q^{3}$$

where *y*_3_ is the vehicle speed (km/h), and *q* is the illuminance change index. The fitting determination coefficient *R*^2^ is 0.95, indicating that the reliability of the fitted model is good.

It can be seen from Figure 7 that the illuminance change index is negatively correlated with the vehicle speed. As the illuminance change index increases, the vehicle speed gradually decreases. In the process of driving, the greater the illumination changes, the more nervous the driver. In consideration of safety, the driver will reduce the driving speed.

### 4.3. Longitudinal Slope Change Model

**Figure 8:**
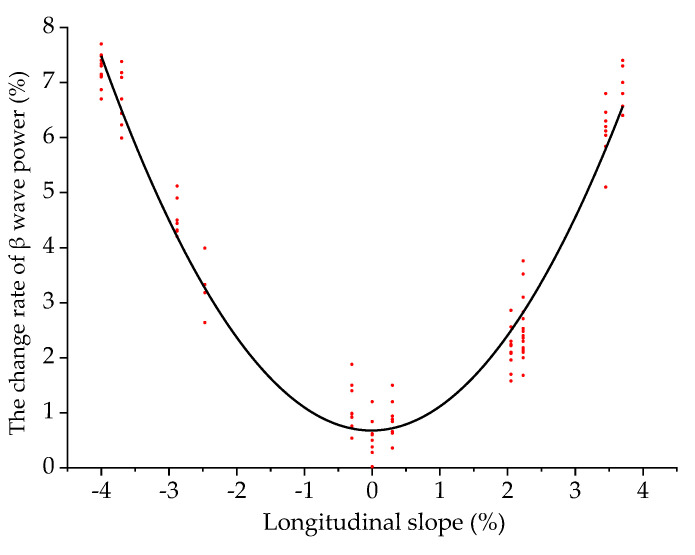
Fitting curve of the longitudinal slope and Δ*P_β_*.

Taking the longitudinal slope as the independent variable and Δ*P_β_* as the dependent variable, the two were fitted. The scatter plot and the fitting curve of the longitudinal slope and Δ*P_β_* are shown in Figure 8.

When a driver is driving in an undersea tunnel, the model of the longitudinal slope and Δ*P_β_* is:

(8) $$y_{1}=0.61-0.24x+0.46x^{2}-0.03x^{3}$$

where *y*_1_ is Δ*P_β_* (%), and *x* is the longitudinal slope (%). The fitting determination coefficient *R*^2^ is 0.97, indicating that the reliability of the fitted model is good.

It can be seen from Figure 8 that the fitting curve of the longitudinal slope and Δ*P_β_* is like a parabola, opening upward. As the longitudinal slope increases, Δ*P_β_* first decreases and then increases, and when the longitudinal slope is −0.1%, Δ*P_β_* reaches the minimum. The fitting curve is approximately symmetrical. The greater the absolute value of the longitudinal slope, the greater the Δ*P_β_*, the more nervous the driver and the worse the driving safety. At the same longitudinal slope, the Δ*P_β_* of the driver in the downslope area is greater than that in the upslope area, which indicates that the driver is more nervous when driving downslope than upslope.

**Figure 9:**
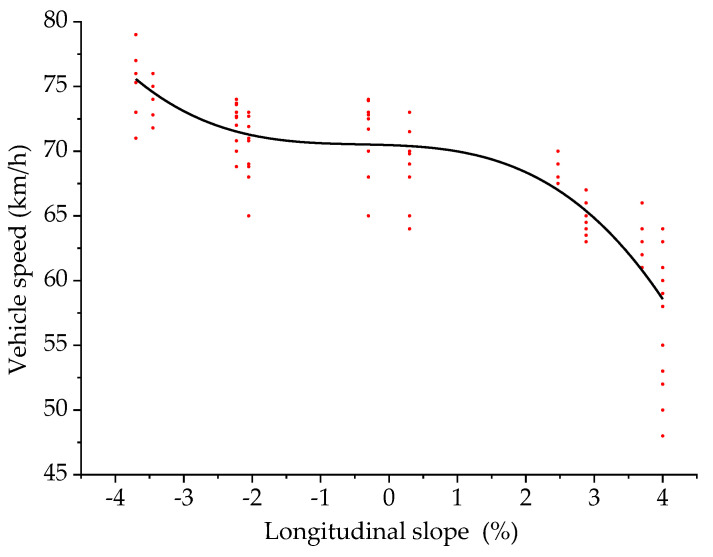
Fitting curve of the longitudinal slope and the vehicle speed.

Taking the longitudinal slope as the independent variable and the vehicle speed as the dependent variable, the two were fitted. The scatter plot and the fitting curve of the longitudinal slope and the vehicle speed are shown in Figure 9.

When a driver is driving in an undersea tunnel, the model of the longitudinal slope and the vehicle speed is:

(9) $$y_{3}=70.50-0.19x-0.17x^{2}-0.13x^{3}$$

where *y*_3_ is vehicle speed (km/h), and *x* is the longitudinal slope (%). The fitting determination coefficient *R*^2^ is 0.78, indicating that the reliability of the fitted model is good.

It can be seen from Figure 9 that the critical points of the model are −2% and 1.5%. When the longitudinal slope is less than −2%, the vehicle speed will decrease rapidly with the increase of the longitudinal slope. When the longitudinal slope is −2% to 1.5%, the vehicle speed is about 71 km/h. When the longitudinal slope is greater than 1.5%, the vehicle speed will decrease rapidly with the increase of the longitudinal slope. There is a certain relationship between the speed change of driving in the tunnel and gravity [37]. When the longitudinal slope is a negative value, it is downslope. The smaller the longitudinal slope value, the greater the longitudinal slope of the downhill, the greater the assistance of gravity to the vehicle and the greater the vehicle speed. When the longitudinal slope is a positive value, it is an upslope. The greater the longitudinal slope value, the greater the longitudinal slope of the upslope, the greater the obstruction of gravity to the vehicle and the lower the vehicle speed.

### 4.4. Parameter Change Model under the Coupling Effect of Illuminance and Longitudinal Slope

Taking the illuminance change index and the longitudinal slope as the independent variable and Δ*P_β_* as the dependent variable, they were fitted. The fitting surface and model of Δ*P_β_* under the coupling effect of the illuminance change index and the longitudinal slope of the undersea tunnel are as follows:

(10) $$y_{1}=0.88+0.26x-0.71q+0.16x^{2}+0.35q^{2}$$

where *y*_1_ is Δ*P_β_* (%), *x* is the longitudinal slope (%) and *q* is the illuminance change index. The fitting determination coefficient *R*^2^ is 0.87, indicating that the reliability of the fitted model is good.

**Figure 10:**
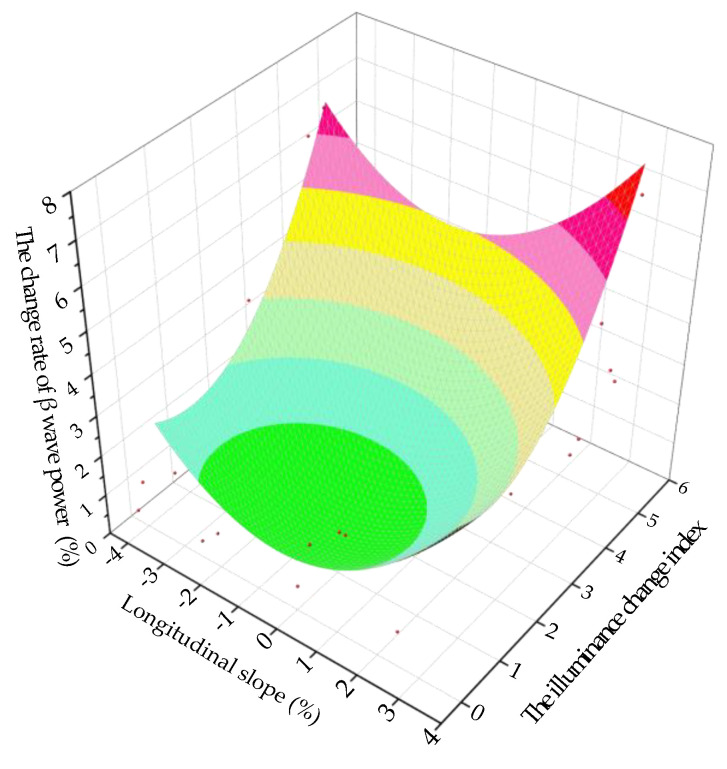
Fitting surface of Δ*P_β_* under the coupling effect of illuminance and longitudinal slope.

It can be seen from Figure 10 that when the illuminance change index is smaller and the absolute value of the longitudinal slope is smaller, Δ*P_β_* is smaller, the psychological pressure of the driver is smaller and the driving safety is better. The greater the illuminance change index and the greater the absolute value of the longitudinal slope, the greater the Δ*P_β_*, the greater the psychological pressure of the driver and the worse the driving safety.

Taking the illuminance change index and the longitudinal slope as the independent variable and the vehicle speed as the dependent variable, they were fitted. The fitting surface and model of the vehicle speed under the coupling effect of the illuminance change index and the longitudinal slope of the undersea tunnel are as follows:

(11) $$y_{3}=85.01-23.42e^{-\frac{x}{8.88}+\frac{q}{6.35}}$$

where *y*_3_ is the vehicle speed (km/h), *x* is the longitudinal slope (%) and *q* is the illuminance change index. The fitting determination coefficient *R*^2^ is 0.93, indicating that the reliability of the fitted model is good.

**Figure 11:**
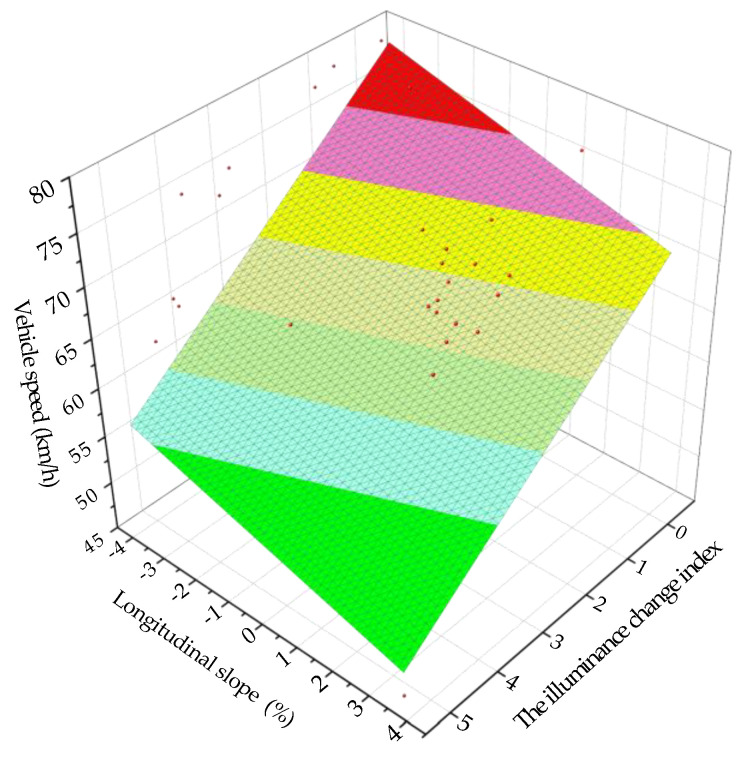
Fitting surface of the vehicle speed under the coupling effect of illuminance and longitudinal slope.

It can be seen from Figure 11 that the greater the illuminance change index and the greater the longitudinal slope, the greater the psychological pressure of the driver and the lower the vehicle speed. The smaller the illuminance change index and the smaller the longitudinal gradient, the smaller the psychological pressure of the driver and the higher the vehicle speed.

### 4.5. Driving Safety Evaluation of the Undersea Tunnel

The occurrence of traffic accidents is closely related to the vehicle speed and the physiological characteristics of the driver [38]. Based on statistical related theory, combined with the driver’s EEG characteristics and speed characteristics during the driving process of the undersea tunnel, the driving safety evaluation model of the undersea tunnel was established as follows:

(12) $$S=f(w,x_{i}),(i=1,2,\cdots ,n)$$

where *S* is the driving safety coefficient (safety comprehensive evaluation coefficient), *S*∈ [0, 1]: the higher the safety comprehensive evaluation score, the better the driving safety, and the lower the safety comprehensive evaluation score, the worse the driving safety. *w* is the index weight vector, and *x_i_* is the evaluation value vector of the *i*-th evaluation object.

Firstly, the traffic safety evaluation index set was constructed as follows:

(13) $$A=\left\{\mathrm{EEG}\;\mathrm{characteristics},\;\mathrm{speed}\;\mathrm{characteristics}\right\}=\left\{\Delta P_{\beta },\Delta P_{(\theta +\alpha )/\beta },\;\mathrm{vehicle}\;\mathrm{speed},\;\mathrm{acceleration}\right\}$$

Before determining the index weight, each evaluation index needs to be standardized. The original evaluation index includes a positive index and inverse index. Only by ensuring the consistency of the evaluation index can the final comprehensive results be used as an evaluation standard, that is, the comprehensive evaluation value of each evaluation object is the larger the better or the smaller the better. The standardized processing model of the evaluation index is as follows:

(14) $$x^{*}{}_{ij}=\frac{1}{\max\left|x_{ij}\right|+x_{ij}}$$

where x*_ij_ is the positive index value generated after the inverse index conversion of each evaluation object.

There is a big difference in the measurement unit and magnitude between the evaluation indicators, so it is necessary to remove the influence of the measurement unit and magnitude through dimensionless processing. The dimensionless model is as follows:

(15) $$z_{ij}=\frac{x_{ij}-\bar{x_{j}}}{\sigma_{j}},(i=1,2,\cdots ,n;j=1,2,\cdots ,m)$$

where $\bar{x_{j}}$ is the mean value of the *j*-th index, and *σ_j_* is the standard deviation of the *j*-th indicator.

Next, to determine the weight of each indicator by the coefficient of variation method, the model is as follows:

(16) $$\bar{x_{j}}=\frac{\sum_{i=1}^{n}x_{ij}}{n}$$

(17) $$\sigma_{j}=\sqrt{\frac{\sum_{i=1}^{n}{\left(x_{ij}-\bar{x_{j}}\right)}^{2}}{n}}$$

(18) $$V_{j}=\frac{\sigma_{j}}{\bar{x_{j}}}$$

(19) $$w_{j}=\frac{V_{j}}{\sum_{i=1}^{n}V_{j}}$$

where *V_j_* is the variation coefficient of the *j*-th index, and *w_j_* is the weight coefficient of the *j*-th index.

**Figure 12:**
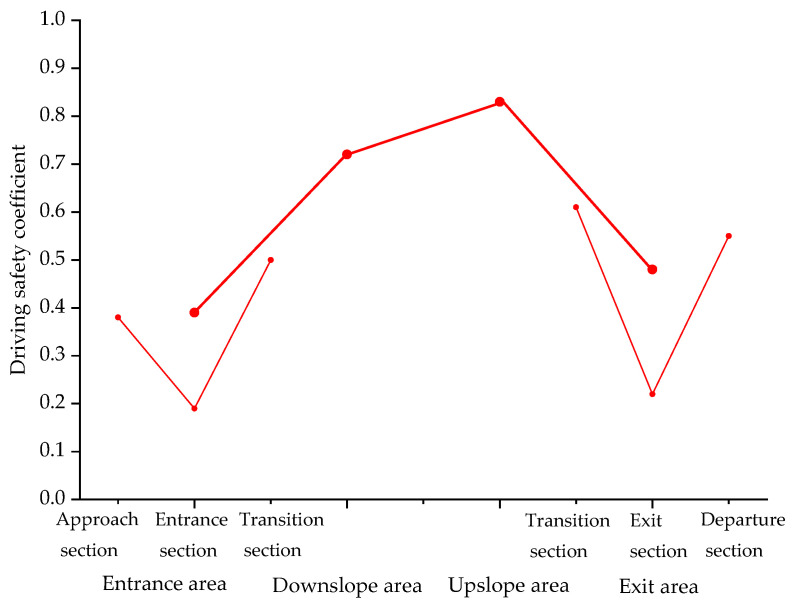
Variation of driving safety coefficient in different areas of the undersea tunnel.

The driving safety coefficient in different areas of the undersea tunnel was calculated, and the results are presented in Figure 12.

It can be seen from Table 2 and Figure 11 that the driving safety coefficient of the undersea tunnel, from high to low, was: upslope area, downslope area, exit area and entrance area, and the driving safety was the same. In the entrance area of the undersea tunnel, the driving safety of the transition section was the best, the approach section was the second and the entrance section was the worst. In the exit area of the undersea tunnel, the driving safety of the transition section was the best, the departure section was the second and the exit section was the worst.

## 5. Discussion

A previous study [23] showed that the longitudinal slope is closely related to traffic safety, and there is no significant difference between the driver’s feeling of downslope and upslope. The results of the present study indicated that the impact of downslope and upslope on drivers is different. Compared with upslope, drivers were more nervous in downslope areas, and the driving safety in upslope areas was slightly better than that in downslope areas. The reason for this difference may be that the research object of the previous study [23] is an ordinary urban tunnel, and most ordinary urban tunnels are medium and short tunnels, and the longitudinal slope is relatively simple, while the research object of this study was a V-shaped undersea tunnel, which is a super long tunnel and has complex longitudinal slope changes throughout the tunnel. This makes the driver more sensitive to the feeling of the longitudinal slope. In addition, the maximum speed limit of the urban tunnel is generally 40–60 km/h, while the maximum speed limit of the Jiaozhou Bay undersea tunnel in this study is 80 km/h. The speed in the undersea tunnel is significantly higher than that in urban tunnels. Different speeds may also affect the driver’s perception of downslope and upslope [14].

The present study found that the traffic safety in the entrance area of the undersea tunnel is poor. This is related to the drastically changing light environment at the entrance of the undersea tunnel. The greater the illumination difference inside and outside the undersea tunnel, the worse the driving safety. The research in [2] also proves this view. In addition, undersea tunnels have complex longitudinal slopes in the entrance area. Affected by the light environment and longitudinal slope, it will reduce the driver’s perception of the driving environment and induce traffic accidents [13].

To further study the impact of the undersea tunnel driving environment on driving safety, the following issues need to be further discussed:

1. Drivers of different genders, age groups, and levels of driving experience may have different perceptions of the undersea tunnel driving environment. Therefore, further research is needed on different driver types.
2. The direction of the tunnel entrance will have an important relationship with the illumination changes at the entrance of the undersea tunnel. If the direction of the entrance of an undersea tunnel is different from that of the Jiaozhou Bay Tunnel, the situation or law may be different from the results of this study.
3. The illuminance factor is only a part of the tunnel light environment. The size, shape, location, and arrangement of the lighting facilities in the undersea tunnel have different effects on the driver’s perception of the light environment. Therefore, it is necessary to further study the influence of tunnel light environment on drivers’ EEG signals and traffic safety.

## 6. Conclusions

In this study, a real vehicle experiment was carried out in Jiaozhou Bay Tunnel to analyze the driving environment of the undersea tunnel and to study the EEG signal and speed characteristics of drivers passing through Jiaozhou Bay Tunnel. The conclusions are as follows:

1. In the entrance and exit area of the undersea tunnel, the illumination, the change rate of EEG power and the vehicle speed have changed significantly, among which the illumination has been reduced by 700 times.
2. Illuminance change index was positively correlated with the change rate of EEG power. The greater the illumination change index, the greater the change rate of EEG power and the more nervous the driver. Illumination change index was negatively correlated with vehicle speed. The greater the illuminance change, the lower the vehicle speed.
3. The absolute value of the longitudinal slope was positively correlated with Δ*P_β_*. The greater the absolute value of the longitudinal slope, the greater the Δ*P_β_*. Under the same longitudinal slope, the drivers were more nervous when driving downslope than upslope. When the longitudinal slope is less than −2% or more than 1.5%, the vehicle speed will decrease with the increase of the longitudinal slope. When the longitudinal slope is between −2% and 1.5%, the vehicle speed is stable at about 71 km/h.
4. It is an approximately opposite and symmetrical process for the driver to enter the entrance area of the undersea tunnel and leave the exit area. The closer to the entrance and exit of the undersea tunnel, the greater the change of the driver’s EEG power, the more nervous the driver and the worse the driving safety.
5. Throughout the undersea tunnel, the safety of the entrance area was the worst, and that of the upslope area was the best. In the entrance area of the undersea tunnel, the safety of the entrance section was the worst.

## Figures and Tables

**Table 1:** Correlation analysis results.

Variable 1	Variable 2	Entrance Area		Downslope Area		Upslope Area		Export Area	
		Correlation	Degree of Relevance	Correlation	Degree of Relevance	Correlation	Degree of Relevance	Correlation	Degree of Relevance
Δ*E*	Δ*P_β_*	0.91	High	—	—	—	—	0.84	High
	Δ*P*_(*θ*+*α*)/*β*_	0.74	Medium	—	—	—	—	0.69	Medium
	Vehicle speed	0.77	Medium	—	—	—	—	0.62	Medium
	Acceleration	0.33	Low	—	—	—	—	0.41	Low
Longitudinal slope	Δ*P_β_*	0.86	High	0.81	High	0.54	Medium	0.83	High
	Δ*P*_(*θ*+*α*)/*β*_	0.65	Medium	0.71	Medium	0.49	Low	0.52	Medium
	Vehicle speed	0.63	Medium	0.51	Medium	0.57	Medium	0.66	Medium
	Acceleration	0.54	Medium	—	—	0.36	Low	0.46	Low

Note: “—” indicates that there is no correlation.

**Table 2:** Driving safety coefficient in different areas of the undersea tunnel.

Position	Entrance Area			Downslope Area	Upslope Area	Exit Area		
	Approach Section	Entrance Section	Transition Section			Transition Section	Exit Section	Departure Section
Driving safety coefficient	0.39			0.72	0.83	0.48		
	0.38	0.20	0.50			0.61	0.23	0.55

## Data Availability

Restrictions apply to the availability of these data. Data were obtained from a real vehicle experiment conducted by the School of Mechanical and Automotive Engineering, Qingdao University of Technology, and are available from the authors with the permission of the School of Mechanical and Automotive Engineering.
